# Supplementary material for: Oral health profiles in the population of older adults in Ecuador: An analysis of latent classes
Source: PLoS One. 2025 Sep 11;20(9):e0330351. doi: 10.1371/journal.pone.0330351 (PMC12425276; doi:10.1371/journal.pone.0330351)
Supplement: Appendix 1 — (DOCX) [file pone.0330351.s001.docx]

**Appendix 1**. *Predicted Probabilities of Classes Based on Multinomial Logistic Regression*

| **Class** | 1 | 2 | 3 | 4 | 5 | 6 | 7 | 8 |
| --- | --- | --- | --- | --- | --- | --- | --- | --- |
| **Variable** | People with no original teeth, complete dentures, minimal problems, and high satisfaction. | People with many missing teeth, dentures, minimal problems, and high satisfaction. | People with few or no missing teeth, some problems, but relatively satisfied | People with many missing teeth, no dentures, moderate problems, and high satisfaction. | People missing many teeth, some with dentures, moderate problems and relatively satisfied. | People with all teeth missing, wearing dentures, significant problems, and high satisfaction. | People with many missing teeth, dentures, significant problems and relatively satisfaction. | People with some missing teeth, no dentures, significant problems, and low satisfaction. |
| Age in categories |  |  |  |  |  |  |  |  |
| 60 to 69 years | 0.163 | 0.244 | 0.171 | 0.117 | 0.085 | 0.087 | 0.061 | 0.071 |
| 70 to 79 years | 0.237 | 0.205 | 0.083 | 0.116 | 0.075 | 0.138 | 0.092 | 0.055 |
| 80 to 89 years | 0.314 | 0.169 | 0.056 | 0.094 | 0.038 | 0.211 | 0.077 | 0.042 |
| 90 years and over | 0.320 | 0.188 | 0.000 | 0.156 | 0.049 | 0.222 | 0.042 | 0.023 |
|  |  |  |  |  |  |  |  |  |
| Gender |  |  |  |  |  |  |  |  |
| Female | 0.262 | 0.241 | 0.069 | 0.085 | 0.066 | 0.152 | 0.072 | 0.053 |
| Male | 0.167 | 0.211 | 0.123 | 0.169 | 0.087 | 0.093 | 0.077 | 0.073 |
|  |  |  |  |  |  |  |  |  |
| Area |  |  |  |  |  |  |  |  |
| Urban | 0.196 | 0.252 | 0.103 | 0.116 | 0.083 | 0.113 | 0.077 | 0.061 |
| Rural | 0.245 | 0.204 | 0.080 | 0.126 | 0.069 | 0.138 | 0.074 | 0.066 |
|  |  |  |  |  |  |  |  |  |
| Medicaments |  |  |  |  |  |  |  |  |
| Yes | 0.215 | 0.238 | 0.091 | 0.115 | 0.079 | 0.129 | 0.071 | 0.062 |
| No | 0.217 | 0.204 | 0.098 | 0.143 | 0.069 | 0.100 | 0.099 | 0.069 |
|  |  |  |  |  |  |  |  |  |
| General health status |  |  |  |  |  |  |  |  |
| Excellent | 0.255 | 0.334 | 0.158 | 0.071 | 0.101 | 0.039 | 0.043 | 0.000 |
| Very good | 0.202 | 0.409 | 0.174 | 0.078 | 0.043 | 0.052 | 0.018 | 0.024 |
| Good | 0.213 | 0.286 | 0.103 | 0.148 | 0.064 | 0.097 | 0.046 | 0.043 |
| Regular | 0.211 | 0.216 | 0.087 | 0.108 | 0.087 | 0.122 | 0.088 | 0.080 |
| Bad | 0.198 | 0.172 | 0.077 | 0.124 | 0.059 | 0.165 | 0.091 | 0.114 |
|  |  |  |  |  |  |  |  |  |
| High pressure |  |  |  |  |  |  |  |  |
| Yes | 0.215 | 0.240 | 0.086 | 0.115 | 0.081 | 0.131 | 0.075 | 0.056 |
| No | 0.217 | 0.224 | 0.098 | 0.124 | 0.074 | 0.117 | 0.076 | 0.069 |
|  |  |  |  |  |  |  |  |  |
| Diabetes |  |  |  |  |  |  |  |  |
| Yes | 0.214 | 0.226 | 0.081 | 0.141 | 0.074 | 0.114 | 0.074 | 0.077 |
| No | 0.216 | 0.232 | 0.095 | 0.118 | 0.077 | 0.125 | 0.076 | 0.061 |
|  |  |  |  |  |  |  |  |  |
| Heart problems |  |  |  |  |  |  |  |  |
| Yes | 0.185 | 0.256 | 0.110 | 0.116 | 0.059 | 0.117 | 0.082 | 0.076 |
| No | 0.220 | 0.228 | 0.091 | 0.121 | 0.080 | 0.124 | 0.075 | 0.062 |
|  |  |  |  |  |  |  |  |  |
| Arthritis, rheumatism or arthrosis |  |  |  |  |  |  |  |  |
| Yes | 0.214 | 0.216 | 0.094 | 0.097 | 0.089 | 0.141 | 0.072 | 0.077 |
| No | 0.216 | 0.237 | 0.092 | 0.132 | 0.072 | 0.116 | 0.077 | 0.058 |
|  |  |  |  |  |  |  |  |  |
| Osteoporosis |  |  |  |  |  |  |  |  |
| Yes | 0.224 | 0.245 | 0.086 | 0.098 | 0.070 | 0.131 | 0.090 | 0.055 |
| No | 0.214 | 0.228 | 0.094 | 0.126 | 0.078 | 0.121 | 0.073 | 0.065 |
|  |  |  |  |  |  |  |  |  |
| Alcohol |  |  |  |  |  |  |  |  |
| I did not consume | 0.224 | 0.222 | 0.089 | 0.123 | 0.081 | 0.119 | 0.075 | 0.066 |
| Less than 1 day per week | 0.179 | 0.241 | 0.094 | 0.083 | 0.078 | 0.158 | 0.094 | 0.072 |
| 1 day a week | 0.172 | 0.263 | 0.123 | 0.138 | 0.089 | 0.065 | 0.100 | 0.050 |
| 2 to 3 days a week | 0.076 | 0.235 | 0.069 | 0.175 | 0.119 | 0.071 | 0.173 | 0.082 |
| 4 to 6 days a week | 0.086 | 0.232 | 0.044 | 0.204 | 0.089 | 0.070 | 0.087 | 0.188 |
| Every day | 0.466 | 0.154 | 0.106 | 0.173 | 0.000 | 0.102 | 0.000 | 0.000 |
|  |  |  |  |  |  |  |  |  |
| Tobacco |  |  |  |  |  |  |  |  |
| I currently smoke | 0.228 | 0.296 | 0.063 | 0.102 | 0.066 | 0.111 | 0.074 | 0.061 |
| I used to smoke but not anymore | 0.208 | 0.252 | 0.074 | 0.123 | 0.081 | 0.129 | 0.082 | 0.051 |
| I have never smoked | 0.216 | 0.211 | 0.111 | 0.122 | 0.077 | 0.122 | 0.072 | 0.070 |
|  |  |  |  |  |  |  |  |  |
| Mental diseases |  |  |  |  |  |  |  |  |
| Yes | 0.188 | 0.203 | 0.102 | 0.109 | 0.081 | 0.151 | 0.084 | 0.082 |
| No | 0.219 | 0.235 | 0.092 | 0.122 | 0.076 | 0.120 | 0.075 | 0.061 |
|  |  |  |  |  |  |  |  |  |
| Province |  |  |  |  |  |  |  |  |
| Azuay | 0.490 | 0.105 | 0.022 | 0.091 | 0.055 | 0.160 | 0.058 | 0.020 |
| Bolívar | 0.325 | 0.168 | 0.097 | 0.032 | 0.062 | 0.159 | 0.032 | 0.124 |
| Cañar | 0.209 | 0.128 | 0.048 | 0.162 | 0.032 | 0.221 | 0.094 | 0.105 |
| Carchi | 0.578 | 0.031 | 0.062 | 0.114 | 0.022 | 0.156 | 0.022 | 0.016 |
| Cotopaxi | 0.187 | 0.097 | 0.091 | 0.084 | 0.073 | 0.226 | 0.081 | 0.160 |
| Chimborazo | 0.150 | 0.087 | 0.129 | 0.108 | 0.104 | 0.149 | 0.072 | 0.202 |
| El Oro | 0.351 | 0.291 | 0.054 | 0.097 | 0.065 | 0.063 | 0.065 | 0.014 |
| Esmeraldas | 0.163 | 0.232 | 0.178 | 0.125 | 0.109 | 0.072 | 0.083 | 0.038 |
| Guayas | 0.140 | 0.350 | 0.106 | 0.126 | 0.080 | 0.081 | 0.069 | 0.049 |
| Imbabura | 0.342 | 0.147 | 0.058 | 0.086 | 0.034 | 0.212 | 0.073 | 0.048 |
| Loja | 0.497 | 0.189 | 0.023 | 0.083 | 0.053 | 0.087 | 0.042 | 0.025 |
| Los Ríos | 0.175 | 0.308 | 0.106 | 0.112 | 0.074 | 0.094 | 0.068 | 0.063 |
| Manabí | 0.081 | 0.255 | 0.130 | 0.221 | 0.080 | 0.062 | 0.064 | 0.107 |
| Pichincha | 0.208 | 0.181 | 0.109 | 0.103 | 0.076 | 0.166 | 0.087 | 0.071 |
| Tungurahua | 0.232 | 0.140 | 0.081 | 0.025 | 0.064 | 0.238 | 0.083 | 0.137 |
