# Supplementary material for: Oral health profiles in the population of older adults in Ecuador: An analysis of latent classes
Source: PLoS One. 2025 Sep 11;20(9):e0330351. doi: 10.1371/journal.pone.0330351 (PMC12425276; doi:10.1371/journal.pone.0330351)
Supplement: Appendix 2 — (DOCX) [file pone.0330351.s002.docx]

**Appendix 2.** *Marginal probabilities by each class.*

| **Class** | **1**  People with no original teeth, complete dentures, minimal problems, and high satisfaction. | **2**  People with many missing teeth, dentures, minimal problems, and high satisfaction. | **3**  People with few or no missing teeth, some problems, but relatively satisfied. | **4**  People with many missing teeth, no dentures, moderate problems, and high satisfaction. | **5**  People missing many teeth, some with dentures, moderate problems, tooth sensitivity and relatively satisfied. | **6**  People with all teeth missing, wearing dentures, significant problems and high satisfaction. | **7**  People with many missing teeth, some wear dentures, significant problems and relatively satisfaction. | **8**  People with many missing teeth, no dentures, significant problems., tooth sensitivity and low satisfaction. |
| --- | --- | --- | --- | --- | --- | --- | --- | --- |
| **Class Marginal Probability** | 0.22 | 0.19 | 0.11 | 0.10 | 0.08 | 0.12 | 0.08 | 0.10 |
| **Statements** |  |  |  |  |  |  |  |  |
| Having all teeth or missing a few teeth | 0.00 | 0.00 | 1.00 | 0.00 | 0.00 | 0.00 | 0.03 | 0.09 |
| Missing many teeth | 0.00 | 0.89 | 0.00 | 0.97 | 1.00 | 0.00 | 0.86 | 0.85 |
| Missing all teeth | 1.00 | 0.11 | 0.00 | 0.03 | 0.00 | 1.00 | 0.11 | 0.06 |
| Not wearing dentures | 0.00 | 0.00 | 0.66 | 1.00 | 0.51 | 0.20 | 0.51 | 0.62 |
| Wearing dentures | 1.00 | 1.00 | 0.23 | 0.00 | 0.49 | 0.80 | 0.49 | 0.38 |
| Having full teeth | 0.00 | 0.00 | 0.11 | 0.00 | 0.00 | 0.00 | 0.00 | 0.00 |
| Changing meals for problems with teeth | 0.07 | 0.07 | 0.07 | 0.14 | 0.30 | 0.66 | 0.67 | 0.74 |
| Having trouble chewing hard food | 0.22 | 0.22 | 0.11 | 0.26 | 0.55 | 0.86 | 0.94 | 0.93 |
| Having trouble speaking well | 0.04 | 0.05 | 0.03 | 0.06 | 0.12 | 0.41 | 0.43 | 0.57 |
| Having trouble eating due to tooth discomfort | 0.08 | 0.10 | 0.04 | 0.12 | 0.34 | 0.77 | 0.82 | 0.93 |
| Having trouble going out or talking to others | 0.02 | 0.01 | 0.00 | 0.02 | 0.05 | 0.27 | 0.31 | 0.47 |
| Being happy when looking in the mirror | 0.68 | 0.68 | 0.72 | 0.48 | 0.56 | 0.56 | 0.41 | 0.39 |
| Using medications to relieve dental pain | 0.03 | 0.05 | 0.07 | 0.07 | 0.25 | 0.24 | 0.24 | 0.41 |
| Concerning about dental status | 0.09 | 0.30 | 0.22 | 0.36 | 0.60 | 0.63 | 0.74 | 0.92 |
| Being nervous about dental problems | 0.02 | 0.08 | 0.07 | 0.09 | 0.23 | 0.39 | 0.44 | 0.69 |
| Having trouble eating in front of others | 0.04 | 0.06 | 0.03 | 0.07 | 0.21 | 0.50 | 0.52 | 0.77 |
| Rarely having dental discomfort due to food temperature or sweetness | 0.00 | 0.96 | 0.81 | 0.98 | 0.00 | 0.00 | 0.91 | 0.00 |
| Frequently having dental discomfort due to food temperature or sweetness | 0.00 | 0.00 | 0.17 | 0.00 | 1.00 | 0.00 | 0.00 | 1.00 |
| Not knowing dental discomfort due to food temperature or sweetness | 1.00 | 0.04 | 0.02 | 0.02 | 0.00 | 1.00 | 0.09 | 0.00 |
