# Supplementary material for: Oral health profiles in the population of older adults in Ecuador: An analysis of latent classes
Source: PLoS One. 2025 Sep 11;20(9):e0330351. doi: 10.1371/journal.pone.0330351 (PMC12425276; doi:10.1371/journal.pone.0330351)
Supplement: Appendix 3 — (DOCX) [file pone.0330351.s003.docx]

**Appendix 3.** *Multinomial Logistic Regression.*

| **Class** | **RRR** | **Std. Error** | **z** | **P>z** | **[95% conf. interval]** | |
| --- | --- | --- | --- | --- | --- | --- |
| **1** | (Base outcome) | |  |  |  |  |
| **2** |  |  |  |  |  |  |
| Age in categories |  |  |  |  |  |  |
| 70 to 79 years | 0.579 | 0.069 | -4.610 | 0.000 | 0.459 | 0.730 |
| 80 to 89 years | 0.359 | 0.059 | -6.240 | 0.000 | 0.260 | 0.496 |
| 90 years and over | 0.393 | 0.151 | -2.440 | 0.015 | 0.186 | 0.833 |
|  |  |  |  |  |  |  |
| Gender |  |  |  |  |  |  |
| Male | 1.369 | 0.191 | 2.250 | 0.025 | 1.041 | 1.800 |
|  |  |  |  |  |  |  |
| Area |  |  |  |  |  |  |
| Rural | 0.649 | 0.074 | -3.790 | 0.000 | 0.519 | 0.812 |
|  |  |  |  |  |  |  |
| Medicaments |  |  |  |  |  |  |
| Sí | 1.173 | 0.169 | 1.100 | 0.270 | 0.884 | 1.557 |
|  |  |  |  |  |  |  |
| General health status |  |  |  |  |  |  |
| Very good | 1.550 | 0.857 | 0.790 | 0.428 | 0.524 | 4.579 |
| Good | 1.022 | 0.488 | 0.050 | 0.963 | 0.401 | 2.606 |
| Regular | 0.781 | 0.369 | -0.520 | 0.602 | 0.309 | 1.973 |
| Bad | 0.661 | 0.323 | -0.850 | 0.398 | 0.254 | 1.723 |
|  |  |  |  |  |  |  |
| High pressure |  |  |  |  |  |  |
| No | 0.928 | 0.106 | -0.650 | 0.513 | 0.742 | 1.160 |
|  |  |  |  |  |  |  |
| Diabetes |  |  |  |  |  |  |
| No | 1.015 | 0.163 | 0.090 | 0.926 | 0.741 | 1.391 |
|  |  |  |  |  |  |  |
| Heart problems |  |  |  |  |  |  |
| No | 0.746 | 0.126 | -1.730 | 0.083 | 0.536 | 1.039 |
|  |  |  |  |  |  |  |
| Arthritis, rheumatism or arthrosis |  |  |  |  |  |  |
| No | 1.090 | 0.136 | 0.690 | 0.492 | 0.853 | 1.392 |
|  |  |  |  |  |  |  |
| Osteoporosis |  |  |  |  |  |  |
| No | 0.973 | 0.144 | -0.180 | 0.855 | 0.728 | 1.301 |
|  |  |  |  |  |  |  |
| BMI | 0.997 | 0.005 | -0.520 | 0.603 | 0.988 | 1.007 |
|  |  |  |  |  |  |  |
| Alcohol |  |  |  |  |  |  |
| Less than 1 day per week | 1.363 | 0.216 | 1.950 | 0.051 | 0.998 | 1.860 |
| 1 day a week | 1.553 | 0.464 | 1.470 | 0.140 | 0.865 | 2.789 |
| 2 to 3 days a week | 3.121 | 1.674 | 2.120 | 0.034 | 1.091 | 8.931 |
| 4 to 6 days a week | 2.744 | 2.795 | 0.990 | 0.322 | 0.372 | 20.212 |
| Every day | 0.334 | 0.254 | -1.440 | 0.150 | 0.075 | 1.484 |
|  |  |  |  |  |  |  |
| Tobacco |  |  |  |  |  |  |
| I used to smoke but not anymore | 0.933 | 0.180 | -0.360 | 0.721 | 0.640 | 1.361 |
| I have never smoked | 0.751 | 0.148 | -1.450 | 0.148 | 0.510 | 1.107 |
|  |  |  |  |  |  |  |
| Dentist attention |  |  |  |  |  |  |
| No | 0.380 | 0.070 | -5.260 | 0.000 | 0.265 | 0.545 |
|  |  |  |  |  |  |  |
| Reasons for not going to the dentist |  |  |  |  |  |  |
| Dentist is far | 0.897 | 0.319 | -0.310 | 0.759 | 0.447 | 1.800 |
| I couldn't pay | 1.321 | 0.242 | 1.520 | 0.128 | 0.923 | 1.891 |
| I had no insurance | 0.448 | 0.649 | -0.550 | 0.579 | 0.026 | 7.656 |
| I have had no one to take me | 0.453 | 0.278 | -1.290 | 0.197 | 0.136 | 1.509 |
| Other | 0.631 | 0.101 | -2.890 | 0.004 | 0.461 | 0.862 |
| I have been to the dentist | 1.000 | (omitted) |  |  |  |  |
|  |  |  |  |  |  |  |
| Mental diseases |  |  |  |  |  |  |
| No | 0.990 | 0.180 | -0.060 | 0.956 | 0.693 | 1.414 |
|  |  |  |  |  |  |  |
| Province |  |  |  |  |  |  |
| Bolívar | 2.417 | 1.177 | 1.810 | 0.070 | 0.930 | 6.279 |
| Cañar | 2.872 | 1.275 | 2.380 | 0.017 | 1.204 | 6.855 |
| Carchi | 0.255 | 0.198 | -1.760 | 0.078 | 0.056 | 1.168 |
| Cotopaxi | 2.434 | 1.041 | 2.080 | 0.038 | 1.053 | 5.629 |
| Chimborazo | 2.716 | 1.272 | 2.130 | 0.033 | 1.085 | 6.799 |
| El Oro | 3.879 | 1.194 | 4.400 | 0.000 | 2.122 | 7.090 |
| Esmeraldas | 6.684 | 2.659 | 4.770 | 0.000 | 3.064 | 14.578 |
| Guayas | 11.715 | 3.093 | 9.320 | 0.000 | 6.982 | 19.657 |
| Imbabura | 2.010 | 0.703 | 2.000 | 0.046 | 1.012 | 3.991 |
| Loja | 1.781 | 0.542 | 1.900 | 0.058 | 0.981 | 3.234 |
| Los Ríos | 8.254 | 2.980 | 5.850 | 0.000 | 4.067 | 16.750 |
| Manabí | 14.821 | 4.666 | 8.560 | 0.000 | 7.996 | 27.468 |
| Pichincha | 4.068 | 1.121 | 5.090 | 0.000 | 2.370 | 6.981 |
| Tungurahua | 2.818 | 1.006 | 2.900 | 0.004 | 1.400 | 5.674 |
|  |  |  |  |  |  |  |
| Constant | 1.161 | 0.770 | 0.230 | 0.822 | 0.316 | 4.261 |
| **3** |  |  |  |  |  |  |
| Age in categories |  |  |  |  |  |  |
| 70 to 79 years | 0.333 | 0.048 | -7.590 | 0.000 | 0.251 | 0.443 |
| 80 to 89 years | 0.170 | 0.039 | -7.810 | 0.000 | 0.109 | 0.265 |
| 90 years and over | 0.000 | 0.000 | -0.020 | 0.982 | 0.000 | . |
|  |  |  |  |  |  |  |
| Gender |  |  |  |  |  |  |
| Male | 2.824 | 0.456 | 6.430 | 0.000 | 2.059 | 3.875 |
|  |  |  |  |  |  |  |
| Area |  |  |  |  |  |  |
| Rural | 0.619 | 0.083 | -3.560 | 0.000 | 0.476 | 0.806 |
|  |  |  |  |  |  |  |
| Medicaments |  |  |  |  |  |  |
| Sí | 0.941 | 0.152 | -0.380 | 0.705 | 0.686 | 1.290 |
|  |  |  |  |  |  |  |
| General health status |  |  |  |  |  |  |
| Very good | 1.392 | 0.813 | 0.570 | 0.571 | 0.443 | 4.372 |
| Good | 0.782 | 0.392 | -0.490 | 0.624 | 0.292 | 2.090 |
| Regular | 0.661 | 0.328 | -0.840 | 0.404 | 0.250 | 1.746 |
| Bad | 0.623 | 0.322 | -0.910 | 0.361 | 0.226 | 1.718 |
|  |  |  |  |  |  |  |
| High pressure |  |  |  |  |  |  |
| No | 1.131 | 0.153 | 0.910 | 0.364 | 0.868 | 1.474 |
|  |  |  |  |  |  |  |
| Diabetes |  |  |  |  |  |  |
| No | 1.159 | 0.229 | 0.750 | 0.456 | 0.786 | 1.708 |
|  |  |  |  |  |  |  |
| Heart problems |  |  |  |  |  |  |
| No | 0.688 | 0.140 | -1.850 | 0.065 | 0.462 | 1.023 |
|  |  |  |  |  |  |  |
| Arthritis, rheumatism or arthrosis |  |  |  |  |  |  |
| No | 0.971 | 0.145 | -0.190 | 0.846 | 0.725 | 1.301 |
|  |  |  |  |  |  |  |
| Osteoporosis |  |  |  |  |  |  |
| No | 1.149 | 0.212 | 0.750 | 0.452 | 0.800 | 1.648 |
|  |  |  |  |  |  |  |
| BMI | 1.004 | 0.005 | 0.740 | 0.458 | 0.994 | 1.013 |
|  |  |  |  |  |  |  |
| Alcohol |  |  |  |  |  |  |
| Less than 1 day per week | 1.323 | 0.240 | 1.540 | 0.124 | 0.926 | 1.889 |
| 1 day a week | 1.799 | 0.571 | 1.850 | 0.064 | 0.965 | 3.352 |
| 2 to 3 days a week | 2.280 | 1.433 | 1.310 | 0.190 | 0.665 | 7.815 |
| 4 to 6 days a week | 1.287 | 1.726 | 0.190 | 0.851 | 0.093 | 17.848 |
| Every day | 0.571 | 0.435 | -0.730 | 0.462 | 0.128 | 2.544 |
|  |  |  |  |  |  |  |
| Tobacco |  |  |  |  |  |  |
| I used to smoke but not anymore | 1.289 | 0.301 | 1.090 | 0.277 | 0.815 | 2.038 |
| I have never smoked | 1.859 | 0.435 | 2.650 | 0.008 | 1.175 | 2.940 |
|  |  |  |  |  |  |  |
| Dentist attention |  |  |  |  |  |  |
| No | 0.375 | 0.079 | -4.650 | 0.000 | 0.249 | 0.567 |
|  |  |  |  |  |  |  |
| Reasons for not going to the dentist |  |  |  |  |  |  |
| Dentist is far | 0.633 | 0.289 | -1.000 | 0.317 | 0.259 | 1.551 |
| I couldn't pay | 1.310 | 0.285 | 1.240 | 0.214 | 0.855 | 2.006 |
| I had no insurance | 1.020 | 1.492 | 0.010 | 0.989 | 0.058 | 17.928 |
| I have had no one to take me | 0.972 | 0.614 | -0.050 | 0.964 | 0.282 | 3.352 |
| Other | 0.646 | 0.124 | -2.270 | 0.023 | 0.443 | 0.941 |
| I have been to the dentist | 1.000 | (omitted) |  |  |  |  |
|  |  |  |  |  |  |  |
| Mental diseases |  |  |  |  |  |  |
| No | 0.773 | 0.163 | -1.220 | 0.221 | 0.511 | 1.168 |
|  |  |  |  |  |  |  |
| Province |  |  |  |  |  |  |
| Bolívar | 6.716 | 4.155 | 3.080 | 0.002 | 1.997 | 22.579 |
| Cañar | 5.208 | 3.221 | 2.670 | 0.008 | 1.549 | 17.501 |
| Carchi | 2.424 | 1.452 | 1.480 | 0.139 | 0.749 | 7.843 |
| Cotopaxi | 10.921 | 5.686 | 4.590 | 0.000 | 3.936 | 30.298 |
| Chimborazo | 19.364 | 10.211 | 5.620 | 0.000 | 6.889 | 54.429 |
| El Oro | 3.465 | 1.745 | 2.470 | 0.014 | 1.291 | 9.300 |
| Esmeraldas | 24.586 | 12.742 | 6.180 | 0.000 | 8.903 | 67.895 |
| Guayas | 17.052 | 7.231 | 6.690 | 0.000 | 7.427 | 39.150 |
| Imbabura | 3.801 | 1.953 | 2.600 | 0.009 | 1.388 | 10.408 |
| Loja | 1.026 | 0.578 | 0.050 | 0.963 | 0.340 | 3.094 |
| Los Ríos | 13.620 | 7.005 | 5.080 | 0.000 | 4.970 | 37.321 |
| Manabí | 36.182 | 16.542 | 7.850 | 0.000 | 14.769 | 88.644 |
| Pichincha | 11.729 | 5.006 | 5.770 | 0.000 | 5.081 | 27.074 |
| Tungurahua | 7.868 | 3.874 | 4.190 | 0.000 | 2.998 | 20.650 |
|  |  |  |  |  |  |  |
| Constant | 0.194 | 0.154 | -2.070 | 0.038 | 0.041 | 0.915 |
| **4** |  |  |  |  |  |  |
| Age in categories |  |  |  |  |  |  |
| 70 to 79 years | 0.681 | 0.094 | -2.770 | 0.006 | 0.519 | 0.894 |
| 80 to 89 years | 0.416 | 0.081 | -4.510 | 0.000 | 0.284 | 0.609 |
| 90 years and over | 0.680 | 0.275 | -0.950 | 0.341 | 0.307 | 1.503 |
|  |  |  |  |  |  |  |
| Gender |  |  |  |  |  |  |
| Male | 3.124 | 0.508 | 7.010 | 0.000 | 2.272 | 4.296 |
|  |  |  |  |  |  |  |
| Area |  |  |  |  |  |  |
| Rural | 0.868 | 0.114 | -1.070 | 0.284 | 0.671 | 1.124 |
|  |  |  |  |  |  |  |
| Medicaments |  |  |  |  |  |  |
| Sí | 0.811 | 0.127 | -1.340 | 0.180 | 0.597 | 1.101 |
|  |  |  |  |  |  |  |
| General health status |  |  |  |  |  |  |
| Very good | 1.389 | 1.129 | 0.400 | 0.686 | 0.282 | 6.835 |
| Good | 2.474 | 1.747 | 1.280 | 0.200 | 0.620 | 9.876 |
| Regular | 1.828 | 1.284 | 0.860 | 0.390 | 0.461 | 7.240 |
| Bad | 2.227 | 1.591 | 1.120 | 0.262 | 0.549 | 9.031 |
|  |  |  |  |  |  |  |
| High pressure |  |  |  |  |  |  |
| No | 1.070 | 0.145 | 0.500 | 0.618 | 0.821 | 1.395 |
|  |  |  |  |  |  |  |
| Diabetes |  |  |  |  |  |  |
| No | 0.825 | 0.158 | -1.000 | 0.317 | 0.567 | 1.202 |
|  |  |  |  |  |  |  |
| Heart problems |  |  |  |  |  |  |
| No | 0.876 | 0.183 | -0.630 | 0.526 | 0.581 | 1.320 |
|  |  |  |  |  |  |  |
| Arthritis, rheumatism or arthrosis |  |  |  |  |  |  |
| No | 1.340 | 0.209 | 1.870 | 0.061 | 0.987 | 1.819 |
|  |  |  |  |  |  |  |
| Osteoporosis |  |  |  |  |  |  |
| No | 1.341 | 0.271 | 1.450 | 0.146 | 0.903 | 1.992 |
|  |  |  |  |  |  |  |
| BMI | 0.999 | 0.006 | -0.140 | 0.892 | 0.987 | 1.011 |
|  |  |  |  |  |  |  |
| Alcohol |  |  |  |  |  |  |
| Less than 1 day per week | 0.849 | 0.166 | -0.830 | 0.404 | 0.579 | 1.247 |
| 1 day a week | 1.465 | 0.479 | 1.170 | 0.243 | 0.772 | 2.781 |
| 2 to 3 days a week | 4.180 | 2.263 | 2.640 | 0.008 | 1.447 | 12.076 |
| 4 to 6 days a week | 4.340 | 4.403 | 1.450 | 0.148 | 0.594 | 31.693 |
| Every day | 0.678 | 0.453 | -0.580 | 0.560 | 0.183 | 2.511 |
|  |  |  |  |  |  |  |
| Tobacco |  |  |  |  |  |  |
| I used to smoke but not anymore | 1.318 | 0.297 | 1.230 | 0.219 | 0.848 | 2.049 |
| I have never smoked | 1.257 | 0.290 | 1.000 | 0.320 | 0.801 | 1.975 |
|  |  |  |  |  |  |  |
| Dentist attention |  |  |  |  |  |  |
| No | 0.987 | 0.209 | -0.060 | 0.950 | 0.651 | 1.495 |
|  |  |  |  |  |  |  |
| Reasons for not going to the dentist |  |  |  |  |  |  |
| Dentist is far | 0.872 | 0.315 | -0.380 | 0.705 | 0.430 | 1.769 |
| I couldn't pay | 1.412 | 0.268 | 1.820 | 0.069 | 0.973 | 2.050 |
| I had no insurance | 0.790 | 1.154 | -0.160 | 0.872 | 0.045 | 13.823 |
| I have had no one to take me | 0.527 | 0.327 | -1.030 | 0.301 | 0.157 | 1.775 |
| Other | 0.308 | 0.056 | -6.450 | 0.000 | 0.216 | 0.441 |
| I have been to the dentist | 1.000 | (omitted) |  |  |  |  |
|  |  |  |  |  |  |  |
| Mental diseases |  |  |  |  |  |  |
| No | 0.961 | 0.215 | -0.180 | 0.858 | 0.620 | 1.489 |
|  |  |  |  |  |  |  |
| Province |  |  |  |  |  |  |
| Bolívar | 0.538 | 0.431 | -0.770 | 0.439 | 0.112 | 2.590 |
| Cañar | 4.196 | 1.870 | 3.220 | 0.001 | 1.752 | 10.051 |
| Carchi | 1.066 | 0.522 | 0.130 | 0.896 | 0.408 | 2.785 |
| Cotopaxi | 2.418 | 1.114 | 1.920 | 0.055 | 0.980 | 5.965 |
| Chimborazo | 3.906 | 1.845 | 2.880 | 0.004 | 1.548 | 9.857 |
| El Oro | 1.501 | 0.549 | 1.110 | 0.267 | 0.733 | 3.075 |
| Esmeraldas | 4.169 | 1.887 | 3.150 | 0.002 | 1.717 | 10.121 |
| Guayas | 4.870 | 1.423 | 5.420 | 0.000 | 2.747 | 8.634 |
| Imbabura | 1.364 | 0.544 | 0.780 | 0.436 | 0.625 | 2.980 |
| Loja | 0.905 | 0.336 | -0.270 | 0.788 | 0.438 | 1.872 |
| Los Ríos | 3.458 | 1.437 | 2.990 | 0.003 | 1.532 | 7.809 |
| Manabí | 14.796 | 4.934 | 8.080 | 0.000 | 7.697 | 28.444 |
| Pichincha | 2.674 | 0.816 | 3.220 | 0.001 | 1.470 | 4.865 |
| Tungurahua | 0.574 | 0.340 | -0.940 | 0.348 | 0.180 | 1.832 |
|  |  |  |  |  |  |  |
| Constant | 0.079 | 0.072 | -2.810 | 0.005 | 0.014 | 0.464 |
| **5** |  |  |  |  |  |  |
| Age in categories |  |  |  |  |  |  |
| 70 to 79 years | 0.603 | 0.093 | -3.270 | 0.001 | 0.445 | 0.816 |
| 80 to 89 years | 0.234 | 0.060 | -5.630 | 0.000 | 0.141 | 0.388 |
| 90 years and over | 0.295 | 0.172 | -2.100 | 0.036 | 0.094 | 0.923 |
|  |  |  |  |  |  |  |
| Gender |  |  |  |  |  |  |
| Male | 2.077 | 0.383 | 3.960 | 0.000 | 1.447 | 2.981 |
|  |  |  |  |  |  |  |
| Area |  |  |  |  |  |  |
| Rural | 0.662 | 0.101 | -2.700 | 0.007 | 0.491 | 0.893 |
|  |  |  |  |  |  |  |
| Medicaments |  |  |  |  |  |  |
| Sí | 1.158 | 0.222 | 0.770 | 0.444 | 0.795 | 1.688 |
|  |  |  |  |  |  |  |
| General health status |  |  |  |  |  |  |
| Very good | 0.539 | 0.430 | -0.770 | 0.439 | 0.113 | 2.574 |
| Good | 0.755 | 0.473 | -0.450 | 0.653 | 0.221 | 2.577 |
| Regular | 1.047 | 0.644 | 0.070 | 0.941 | 0.313 | 3.498 |
| Bad | 0.757 | 0.483 | -0.440 | 0.663 | 0.217 | 2.641 |
|  |  |  |  |  |  |  |
| High pressure |  |  |  |  |  |  |
| No | 0.903 | 0.136 | -0.670 | 0.500 | 0.672 | 1.214 |
|  |  |  |  |  |  |  |
| Diabetes |  |  |  |  |  |  |
| No | 1.039 | 0.221 | 0.180 | 0.857 | 0.685 | 1.575 |
|  |  |  |  |  |  |  |
| Heart problems |  |  |  |  |  |  |
| No | 1.133 | 0.273 | 0.520 | 0.604 | 0.707 | 1.816 |
|  |  |  |  |  |  |  |
| Arthritis, rheumatism or arthrosis |  |  |  |  |  |  |
| No | 0.803 | 0.130 | -1.360 | 0.175 | 0.585 | 1.103 |
|  |  |  |  |  |  |  |
| Osteoporosis |  |  |  |  |  |  |
| No | 1.164 | 0.235 | 0.750 | 0.452 | 0.784 | 1.729 |
|  |  |  |  |  |  |  |
| BMI | 0.986 | 0.011 | -1.300 | 0.194 | 0.966 | 1.007 |
|  |  |  |  |  |  |  |
| Alcohol |  |  |  |  |  |  |
| Less than 1 day per week | 1.217 | 0.259 | 0.920 | 0.355 | 0.802 | 1.848 |
| 1 day a week | 1.445 | 0.544 | 0.980 | 0.328 | 0.691 | 3.020 |
| 2 to 3 days a week | 4.339 | 2.673 | 2.380 | 0.017 | 1.297 | 14.513 |
| 4 to 6 days a week | 2.880 | 3.761 | 0.810 | 0.418 | 0.223 | 37.217 |
| Every day | 0.000 | 0.000 | -0.010 | 0.993 | 0.000 | . |
|  |  |  |  |  |  |  |
| Tobacco |  |  |  |  |  |  |
| I used to smoke but not anymore | 1.351 | 0.356 | 1.140 | 0.254 | 0.805 | 2.265 |
| I have never smoked | 1.232 | 0.333 | 0.770 | 0.441 | 0.725 | 2.092 |
|  |  |  |  |  |  |  |
| Dentist attention |  |  |  |  |  |  |
| No | 0.294 | 0.071 | -5.100 | 0.000 | 0.184 | 0.471 |
|  |  |  |  |  |  |  |
| Reasons for not going to the dentist |  |  |  |  |  |  |
| Dentist is far | 1.495 | 0.652 | 0.920 | 0.357 | 0.636 | 3.514 |
| I couldn't pay | 2.774 | 0.647 | 4.370 | 0.000 | 1.756 | 4.382 |
| I had no insurance | 0.000 | 0.000 | 0.000 | 0.997 | 0.000 | . |
| I have had no one to take me | 2.205 | 1.238 | 1.410 | 0.159 | 0.733 | 6.628 |
| Other | 0.312 | 0.079 | -4.580 | 0.000 | 0.189 | 0.513 |
| I have been to the dentist | 1.000 | (omitted) |  |  |  |  |
|  |  |  |  |  |  |  |
| Mental diseases |  |  |  |  |  |  |
| No | 0.813 | 0.186 | -0.900 | 0.366 | 0.520 | 1.273 |
|  |  |  |  |  |  |  |
| Province |  |  |  |  |  |  |
| Bolívar | 1.710 | 1.143 | 0.800 | 0.422 | 0.462 | 6.334 |
| Cañar | 1.368 | 0.974 | 0.440 | 0.660 | 0.339 | 5.521 |
| Carchi | 0.335 | 0.275 | -1.330 | 0.182 | 0.067 | 1.671 |
| Cotopaxi | 3.512 | 1.805 | 2.440 | 0.015 | 1.282 | 9.619 |
| Chimborazo | 6.204 | 3.145 | 3.600 | 0.000 | 2.297 | 16.757 |
| El Oro | 1.655 | 0.761 | 1.090 | 0.274 | 0.672 | 4.076 |
| Esmeraldas | 6.033 | 3.072 | 3.530 | 0.000 | 2.224 | 16.368 |
| Guayas | 5.147 | 1.912 | 4.410 | 0.000 | 2.485 | 10.659 |
| Imbabura | 0.904 | 0.506 | -0.180 | 0.856 | 0.302 | 2.706 |
| Loja | 0.963 | 0.454 | -0.080 | 0.937 | 0.383 | 2.425 |
| Los Ríos | 3.800 | 1.862 | 2.720 | 0.006 | 1.454 | 9.930 |
| Manabí | 8.943 | 3.738 | 5.240 | 0.000 | 3.942 | 20.290 |
| Pichincha | 3.259 | 1.254 | 3.070 | 0.002 | 1.533 | 6.927 |
| Tungurahua | 2.490 | 1.193 | 1.900 | 0.057 | 0.973 | 6.368 |
|  |  |  |  |  |  |  |
| Constant | 0.503 | 0.462 | -0.750 | 0.455 | 0.083 | 3.044 |
| **6** |  |  |  |  |  |  |
| Age in categories |  |  |  |  |  |  |
| 70 to 79 years | 1.086 | 0.148 | 0.610 | 0.543 | 0.832 | 1.418 |
| 80 to 89 years | 1.254 | 0.207 | 1.370 | 0.170 | 0.907 | 1.733 |
| 90 years and over | 1.295 | 0.482 | 0.690 | 0.488 | 0.624 | 2.688 |
|  |  |  |  |  |  |  |
| Gender |  |  |  |  |  |  |
| Male | 0.959 | 0.153 | -0.260 | 0.791 | 0.701 | 1.312 |
|  |  |  |  |  |  |  |
| Area |  |  |  |  |  |  |
| Rural | 0.979 | 0.124 | -0.170 | 0.864 | 0.763 | 1.254 |
|  |  |  |  |  |  |  |
| Medicaments |  |  |  |  |  |  |
| Sí | 1.297 | 0.222 | 1.520 | 0.129 | 0.927 | 1.814 |
|  |  |  |  |  |  |  |
| General health status |  |  |  |  |  |  |
| Very good | 1.715 | 2.063 | 0.450 | 0.654 | 0.162 | 18.118 |
| Good | 3.003 | 3.229 | 1.020 | 0.307 | 0.365 | 24.712 |
| Regular | 3.833 | 4.103 | 1.260 | 0.209 | 0.470 | 31.235 |
| Bad | 5.497 | 5.915 | 1.580 | 0.113 | 0.667 | 45.291 |
|  |  |  |  |  |  |  |
| High pressure |  |  |  |  |  |  |
| No | 0.883 | 0.112 | -0.980 | 0.325 | 0.689 | 1.132 |
|  |  |  |  |  |  |  |
| Diabetes |  |  |  |  |  |  |
| No | 1.082 | 0.198 | 0.430 | 0.666 | 0.756 | 1.548 |
|  |  |  |  |  |  |  |
| Heart problems |  |  |  |  |  |  |
| No | 0.885 | 0.164 | -0.660 | 0.509 | 0.615 | 1.273 |
|  |  |  |  |  |  |  |
| Arthritis, rheumatism or arthrosis |  |  |  |  |  |  |
| No | 0.814 | 0.108 | -1.550 | 0.122 | 0.628 | 1.056 |
|  |  |  |  |  |  |  |
| Osteoporosis |  |  |  |  |  |  |
| No | 0.967 | 0.152 | -0.210 | 0.832 | 0.711 | 1.316 |
|  |  |  |  |  |  |  |
| BMI | 0.989 | 0.008 | -1.370 | 0.171 | 0.975 | 1.005 |
|  |  |  |  |  |  |  |
| Alcohol |  |  |  |  |  |  |
| Less than 1 day per week | 1.657 | 0.291 | 2.870 | 0.004 | 1.174 | 2.339 |
| 1 day a week | 0.715 | 0.346 | -0.690 | 0.488 | 0.277 | 1.846 |
| 2 to 3 days a week | 1.761 | 1.305 | 0.760 | 0.445 | 0.412 | 7.524 |
| 4 to 6 days a week | 1.541 | 1.937 | 0.340 | 0.731 | 0.131 | 18.108 |
| Every day | 0.410 | 0.336 | -1.090 | 0.276 | 0.083 | 2.039 |
|  |  |  |  |  |  |  |
| Tobacco |  |  |  |  |  |  |
| I used to smoke but not anymore | 1.268 | 0.317 | 0.950 | 0.342 | 0.777 | 2.071 |
| I have never smoked | 1.153 | 0.292 | 0.560 | 0.574 | 0.702 | 1.895 |
|  |  |  |  |  |  |  |
| Dentist attention |  |  |  |  |  |  |
| No | 0.723 | 0.156 | -1.510 | 0.132 | 0.474 | 1.102 |
|  |  |  |  |  |  |  |
| Reasons for not going to the dentist |  |  |  |  |  |  |
| Dentist is far | 1.595 | 0.550 | 1.350 | 0.176 | 0.812 | 3.135 |
| I couldn't pay | 2.278 | 0.443 | 4.230 | 0.000 | 1.556 | 3.336 |
| I had no insurance | 5.464 | 6.455 | 1.440 | 0.151 | 0.539 | 55.356 |
| I have had no one to take me | 1.934 | 0.862 | 1.480 | 0.139 | 0.808 | 4.632 |
| Other | 0.458 | 0.084 | -4.230 | 0.000 | 0.319 | 0.657 |
| I have been to the dentist | 1.000 | (omitted) |  |  |  |  |
|  |  |  |  |  |  |  |
| Mental diseases |  |  |  |  |  |  |
| No | 0.681 | 0.126 | -2.080 | 0.037 | 0.474 | 0.977 |
|  |  |  |  |  |  |  |
| Province |  |  |  |  |  |  |
| Bolívar | 1.505 | 0.678 | 0.910 | 0.364 | 0.623 | 3.638 |
| Cañar | 3.249 | 1.146 | 3.340 | 0.001 | 1.627 | 6.486 |
| Carchi | 0.828 | 0.351 | -0.450 | 0.656 | 0.361 | 1.900 |
| Cotopaxi | 3.706 | 1.217 | 3.990 | 0.000 | 1.947 | 7.056 |
| Chimborazo | 3.061 | 1.193 | 2.870 | 0.004 | 1.426 | 6.571 |
| El Oro | 0.549 | 0.202 | -1.630 | 0.103 | 0.267 | 1.129 |
| Esmeraldas | 1.367 | 0.652 | 0.650 | 0.513 | 0.536 | 3.483 |
| Guayas | 1.769 | 0.429 | 2.350 | 0.019 | 1.100 | 2.845 |
| Imbabura | 1.905 | 0.569 | 2.160 | 0.031 | 1.061 | 3.421 |
| Loja | 0.538 | 0.166 | -2.010 | 0.044 | 0.294 | 0.985 |
| Los Ríos | 1.661 | 0.639 | 1.320 | 0.187 | 0.781 | 3.530 |
| Manabí | 2.367 | 0.762 | 2.680 | 0.007 | 1.259 | 4.447 |
| Pichincha | 2.443 | 0.591 | 3.690 | 0.000 | 1.520 | 3.924 |
| Tungurahua | 3.152 | 0.970 | 3.730 | 0.000 | 1.724 | 5.761 |
|  |  |  |  |  |  |  |
| Constant | 0.171 | 0.204 | -1.480 | 0.139 | 0.016 | 1.773 |
| **7** |  |  |  |  |  |  |
| Age in categories |  |  |  |  |  |  |
| 70 to 79 years | 1.041 | 0.161 | 0.260 | 0.796 | 0.769 | 1.409 |
| 80 to 89 years | 0.655 | 0.141 | -1.960 | 0.050 | 0.429 | 1.000 |
| 90 years and over | 0.356 | 0.231 | -1.590 | 0.112 | 0.100 | 1.272 |
|  |  |  |  |  |  |  |
| Gender |  |  |  |  |  |  |
| Male | 1.674 | 0.310 | 2.780 | 0.005 | 1.164 | 2.408 |
|  |  |  |  |  |  |  |
| Area |  |  |  |  |  |  |
| Rural | 0.766 | 0.115 | -1.770 | 0.076 | 0.570 | 1.029 |
|  |  |  |  |  |  |  |
| Medicaments |  |  |  |  |  |  |
| Sí | 0.719 | 0.130 | -1.820 | 0.068 | 0.504 | 1.025 |
|  |  |  |  |  |  |  |
| General health status |  |  |  |  |  |  |
| Very good | 0.514 | 0.570 | -0.600 | 0.548 | 0.059 | 4.516 |
| Good | 1.277 | 1.064 | 0.290 | 0.769 | 0.249 | 6.540 |
| Regular | 2.439 | 2.003 | 1.090 | 0.278 | 0.488 | 12.200 |
| Bad | 2.696 | 2.247 | 1.190 | 0.234 | 0.526 | 13.810 |
|  |  |  |  |  |  |  |
| High pressure |  |  |  |  |  |  |
| No | 1.009 | 0.153 | 0.060 | 0.955 | 0.750 | 1.357 |
|  |  |  |  |  |  |  |
| Diabetes |  |  |  |  |  |  |
| No | 1.016 | 0.217 | 0.070 | 0.940 | 0.668 | 1.545 |
|  |  |  |  |  |  |  |
| Heart problems |  |  |  |  |  |  |
| No | 0.765 | 0.168 | -1.220 | 0.222 | 0.497 | 1.176 |
|  |  |  |  |  |  |  |
| Arthritis, rheumatism or arthrosis |  |  |  |  |  |  |
| No | 1.067 | 0.176 | 0.400 | 0.693 | 0.773 | 1.474 |
|  |  |  |  |  |  |  |
| Osteoporosis |  |  |  |  |  |  |
| No | 0.851 | 0.165 | -0.830 | 0.406 | 0.582 | 1.245 |
|  |  |  |  |  |  |  |
| BMI | 0.999 | 0.006 | -0.180 | 0.856 | 0.987 | 1.011 |
|  |  |  |  |  |  |  |
| Alcohol |  |  |  |  |  |  |
| Less than 1 day per week | 1.561 | 0.328 | 2.120 | 0.034 | 1.034 | 2.356 |
| 1 day a week | 1.736 | 0.676 | 1.420 | 0.156 | 0.810 | 3.724 |
| 2 to 3 days a week | 6.769 | 3.979 | 3.250 | 0.001 | 2.138 | 21.424 |
| 4 to 6 days a week | 3.029 | 3.940 | 0.850 | 0.394 | 0.237 | 38.773 |
| Every day | 0.000 | 0.000 | -0.010 | 0.993 | 0.000 | . |
|  |  |  |  |  |  |  |
| Tobacco |  |  |  |  |  |  |
| I used to smoke but not anymore | 1.224 | 0.318 | 0.780 | 0.437 | 0.735 | 2.037 |
| I have never smoked | 1.034 | 0.276 | 0.130 | 0.900 | 0.613 | 1.745 |
|  |  |  |  |  |  |  |
| Dentist attention |  |  |  |  |  |  |
| No | 0.347 | 0.081 | -4.530 | 0.000 | 0.219 | 0.548 |
|  |  |  |  |  |  |  |
| Reasons for not going to the dentist |  |  |  |  |  |  |
| Dentist is far | 0.823 | 0.412 | -0.390 | 0.697 | 0.309 | 2.196 |
| I couldn't pay | 2.327 | 0.523 | 3.760 | 0.000 | 1.498 | 3.615 |
| I had no insurance | 0.000 | 0.001 | 0.000 | 0.997 | 0.000 | . |
| I have had no one to take me | 2.778 | 1.354 | 2.100 | 0.036 | 1.069 | 7.221 |
| Other | 0.263 | 0.065 | -5.410 | 0.000 | 0.162 | 0.427 |
| I have been to the dentist | 1.000 | (omitted) |  |  |  |  |
|  |  |  |  |  |  |  |
| Mental diseases |  |  |  |  |  |  |
| No | 0.767 | 0.175 | -1.170 | 0.243 | 0.491 | 1.198 |
|  |  |  |  |  |  |  |
| Province |  |  |  |  |  |  |
| Bolívar | 0.843 | 0.697 | -0.210 | 0.836 | 0.167 | 4.260 |
| Cañar | 3.770 | 1.970 | 2.540 | 0.011 | 1.354 | 10.500 |
| Carchi | 0.319 | 0.260 | -1.400 | 0.161 | 0.065 | 1.574 |
| Cotopaxi | 3.665 | 1.813 | 2.630 | 0.009 | 1.390 | 9.665 |
| Chimborazo | 4.032 | 2.156 | 2.610 | 0.009 | 1.414 | 11.501 |
| El Oro | 1.550 | 0.697 | 0.970 | 0.330 | 0.642 | 3.741 |
| Esmeraldas | 4.323 | 2.254 | 2.810 | 0.005 | 1.556 | 12.011 |
| Guayas | 4.130 | 1.492 | 3.930 | 0.000 | 2.034 | 8.384 |
| Imbabura | 1.789 | 0.816 | 1.270 | 0.203 | 0.731 | 4.375 |
| Loja | 0.718 | 0.346 | -0.690 | 0.493 | 0.279 | 1.848 |
| Los Ríos | 3.295 | 1.596 | 2.460 | 0.014 | 1.275 | 8.512 |
| Manabí | 6.709 | 2.775 | 4.600 | 0.000 | 2.982 | 15.092 |
| Pichincha | 3.508 | 1.296 | 3.400 | 0.001 | 1.700 | 7.237 |
| Tungurahua | 3.029 | 1.374 | 2.440 | 0.015 | 1.245 | 7.367 |
|  |  |  |  |  |  |  |
| Constant | 0.298 | 0.303 | -1.190 | 0.234 | 0.040 | 2.193 |
| **8** |  |  |  |  |  |  |
| Age in categories |  |  |  |  |  |  |
| 70 to 79 years | 0.536 | 0.080 | -4.170 | 0.000 | 0.400 | 0.719 |
| 80 to 89 years | 0.303 | 0.068 | -5.310 | 0.000 | 0.195 | 0.471 |
| 90 years and over | 0.165 | 0.128 | -2.320 | 0.020 | 0.036 | 0.756 |
|  |  |  |  |  |  |  |
| Gender |  |  |  |  |  |  |
| Male | 2.142 | 0.379 | 4.300 | 0.000 | 1.514 | 3.030 |
|  |  |  |  |  |  |  |
| Area |  |  |  |  |  |  |
| Rural | 0.861 | 0.123 | -1.050 | 0.295 | 0.650 | 1.139 |
|  |  |  |  |  |  |  |
| Medicaments |  |  |  |  |  |  |
| Yes | 0.896 | 0.159 | -0.620 | 0.536 | 0.632 | 1.269 |
|  |  |  |  |  |  |  |
| General health status |  |  |  |  |  |  |
| Very good | 1553678.000 | 1550000000.000 | 0.010 | 0.989 | 0.000 | . |
| Good | 2588223.000 | 2580000000.000 | 0.010 | 0.988 | 0.000 | . |
| Regular | 4907024.000 | 4890000000.000 | 0.020 | 0.988 | 0.000 | . |
| Bad | 7414858.000 | 7390000000.000 | 0.020 | 0.987 | 0.000 | . |
|  |  |  |  |  |  |  |
| High pressure |  |  |  |  |  |  |
| No | 1.217 | 0.177 | 1.350 | 0.176 | 0.916 | 1.619 |
|  |  |  |  |  |  |  |
| Diabetes |  |  |  |  |  |  |
| No | 0.782 | 0.154 | -1.250 | 0.213 | 0.532 | 1.151 |
|  |  |  |  |  |  |  |
| Heart problems |  |  |  |  |  |  |
| No | 0.681 | 0.142 | -1.840 | 0.066 | 0.452 | 1.026 |
|  |  |  |  |  |  |  |
| Arthritis, rheumatism or arthrosis |  |  |  |  |  |  |
| No | 0.744 | 0.113 | -1.950 | 0.051 | 0.553 | 1.002 |
|  |  |  |  |  |  |  |
| Osteoporosis |  |  |  |  |  |  |
| No | 1.241 | 0.237 | 1.130 | 0.257 | 0.854 | 1.805 |
|  |  |  |  |  |  |  |
| BMI | 0.982 | 0.011 | -1.590 | 0.113 | 0.960 | 1.004 |
|  |  |  |  |  |  |  |
| Alcohol |  |  |  |  |  |  |
| Less than 1 day per week | 1.363 | 0.274 | 1.540 | 0.123 | 0.919 | 2.022 |
| 1 day a week | 0.990 | 0.409 | -0.020 | 0.981 | 0.441 | 2.226 |
| 2 to 3 days a week | 3.669 | 2.376 | 2.010 | 0.045 | 1.031 | 13.058 |
| 4 to 6 days a week | 7.447 | 8.494 | 1.760 | 0.078 | 0.796 | 69.639 |
| Every day | 0.000 | 0.000 | -0.010 | 0.991 | 0.000 | . |
|  |  |  |  |  |  |  |
| Tobacco |  |  |  |  |  |  |
| I used to smoke but not anymore | 0.920 | 0.232 | -0.330 | 0.742 | 0.562 | 1.508 |
| I have never smoked | 1.223 | 0.309 | 0.800 | 0.425 | 0.745 | 2.008 |
|  |  |  |  |  |  |  |
| Dentist attention |  |  |  |  |  |  |
| No | 0.365 | 0.082 | -4.470 | 0.000 | 0.234 | 0.567 |
|  |  |  |  |  |  |  |
| Reasons for not going to the dentist |  |  |  |  |  |  |
| Dentist is far | 1.310 | 0.533 | 0.660 | 0.507 | 0.590 | 2.910 |
| I couldn't pay | 3.063 | 0.651 | 5.260 | 0.000 | 2.019 | 4.646 |
| I had no insurance | 2.021 | 2.918 | 0.490 | 0.626 | 0.119 | 34.245 |
| I have had no one to take me | 0.540 | 0.436 | -0.760 | 0.445 | 0.111 | 2.629 |
| Other | 0.202 | 0.050 | -6.410 | 0.000 | 0.124 | 0.329 |
| I have been to the dentist | 1.000 | (omitted) |  |  |  |  |
|  |  |  |  |  |  |  |
| Mental diseases |  |  |  |  |  |  |
| No | 0.642 | 0.135 | -2.110 | 0.035 | 0.425 | 0.970 |
|  |  |  |  |  |  |  |
| Province |  |  |  |  |  |  |
| Bolívar | 9.350 | 5.938 | 3.520 | 0.000 | 2.693 | 32.467 |
| Cañar | 12.324 | 7.254 | 4.270 | 0.000 | 3.888 | 39.064 |
| Carchi | 0.674 | 0.594 | -0.450 | 0.655 | 0.120 | 3.794 |
| Cotopaxi | 20.923 | 11.442 | 5.560 | 0.000 | 7.164 | 61.108 |
| Chimborazo | 33.013 | 18.693 | 6.180 | 0.000 | 10.881 | 100.156 |
| El Oro | 0.957 | 0.673 | -0.060 | 0.950 | 0.241 | 3.802 |
| Esmeraldas | 5.671 | 3.858 | 2.550 | 0.011 | 1.495 | 21.517 |
| Guayas | 8.544 | 4.213 | 4.350 | 0.000 | 3.250 | 22.460 |
| Imbabura | 3.462 | 2.005 | 2.140 | 0.032 | 1.112 | 10.775 |
| Loja | 1.216 | 0.750 | 0.320 | 0.751 | 0.363 | 4.071 |
| Los Ríos | 8.829 | 5.114 | 3.760 | 0.000 | 2.837 | 27.473 |
| Manabí | 32.519 | 16.844 | 6.720 | 0.000 | 11.782 | 89.752 |
| Pichincha | 8.345 | 4.165 | 4.250 | 0.000 | 3.137 | 22.198 |
| Tungurahua | 14.438 | 7.726 | 4.990 | 0.000 | 5.058 | 41.208 |
|  |  |  |  |  |  |  |
| Constant | 0.000 | 0.000 | -0.020 | 0.987 | 0.000 | . |
